# VEGFR1 primes a unique cohort of dental pulp stem cells for vasculogenic differentiation

**DOI:** 10.22203/eCM.v041a21

**Published:** 2021-03-16

**Authors:** M.T. Bergamo, Z. Zhang, T.M. Oliveira, J.E. Nör

**Affiliations:** 1 Department of Pediatric Dentistry, Orthodontics and Collective Health, Bauru School of Dentistry, University of São Paulo, Bauru, SP, Brazil;; 2 Department of Cariology, Restorative Sciences and Endodontics, School of Dentistry, University of Michigan, Ann Arbor, MI, USA

**Keywords:** stem cells, differentiation, angiogenesis, vasculogenesis, pulp biology, multipotency, tissue regeneration, vascular endothelial growth factor, endodontics

## Abstract

Dental Pulp Stem Cells (DPSC) constitute a unique group of cells endowed with multipotency, self-renewal, and the capacity to regenerate the dental pulp tissue. While much has been learned about these cells in recent years, it is still unclear if each DPSC cell is multipotent or if unique sub-populations of DPSCs are “primed” to undergo specific differentiation paths. The purpose of this study was to define whether a sub-population of DPSCs is uniquely primed to undergo vasculogenic differentiation. Here, permanent tooth DPSCs or Stem cells from Human Exfoliated Deciduous teeth (SHED) were flow sorted for VEGFR1 and exposed to vasculogenic differentiation medium, *i.e*. Endothelial Growth Medium (EGM) 2-MV supplemented with 50 ng/ml rhVEGF_165_ in presence of 0 or 25 μg/ml anti-human VEGF antibody (bevacizumab; Genentech). In addition, sorted SHED (*i.e*. VEGFR1^high^ or VEGFR1^low^) were seeded in biodegradable scaffolds and transplanted into the subcutaneous space of immunodeficient mice. Despite proliferating at a similar rate, VEGFR1^high^ generated more *in vitro* sprouts than VEGFR1^low^ cells (p<0.05). Blockade of VEGF signaling with bevacizumab inhibited VEGFR1^high^-derived sprouts, demonstrating specificity of responses. Similarly, VEGFR1^high^ SHED generated more blood vessels when transplanted into murine hosts than VEGFR1^low^ cells (p<0.05). Collectively, these data demonstrate that dental pulp stem cells contain a unique sub-population of cells defined by high VEGFR1 expression that are primed to differentiate into vascular endothelial cells. These data raise the possibility of purifying stem cells with high vasculogenic potential for regeneration of vascularized tissues or for vascular engineering in the treatment of ischemic conditions.

## Graphical Abstract

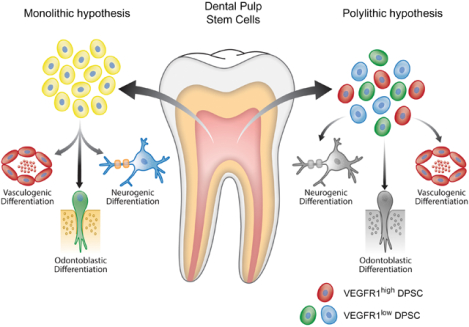

**Schematic representation of the hypothesis tested here.** Drawing highlights 2 working hypotheses for the multipotency of dental pulp stem cells. The monolithic hypothesis suggests that each single dental pulp stem cell is multipotent and capable of undergoing differentiation towards multiple different cell types (e.g. odontoblast, neural cell, endothelial cell). In contrast, the polylithic hypothesis proposes that dental pulp stem cells constitute a heterogeneous group of cells with different sub-populations of cells that are “primed” to a certain differentiation fate. Under this hypothesis, a certain cell sub-group is primed to become an endothelial cell while another sub-group can be primed to become an odontoblast or a neural cell.

## Introduction

Dental pulp tissue regeneration requires the odontogenic and vasculogenic differentiation of resident stem cells (Sakai *et al*., 2010). These differentiation pathways are engaged by dental pulp stem cells (DPSC) in several clinical scenarios, such as direct pulp capping or pulpotomy (Casagrande *et al*., 2011; Fitzgerald *et al*., 1990). Interestingly, similar responses are also observed when DPSCs from permanent teeth (Gronthos *et al*., 2000) or stem cells from exfoliated deciduous teeth (SHED; Miura *et al*., 2003) are transplanted into empty root canals in attempt to engineer a new dental pulp tissue (Casagrande *et al*., 2010; Cordeiro *et al*., 2008; Rosa *et al*., 2013; Smith *et al*., 2016). While it has been clearly demonstrated that pulp stem cells differentiate into functional odontoblasts and vasculogenic endothelial cells (Sakai *et al*., 2010; Xu *et al*., 2019) that regenerate pulp-like tissues (Gan *et al*., 2020; Piva *et al*., 2017; Rosa *et al*., 2013), mechanisms underlying fate decisions of dental pulp stem cells remain elusive. Deep mechanistic understanding of stem cell fate decisions will allow for temporal and spatial control of differentiation events, which may further improve the success of ongoing clinical trials aiming at the engineering of dental pulps for treatment of necrotic teeth (Nakashima *et al*., 2017; Xuan *et al*., 2018).

The phenotypic hallmarks of physiological tissue-specific stem cells are self-renewal and multipotency. Recent studies showed that the presence of pulp stem cells in the perivascular niche (Oh and Nör, 2015) enables their crosstalk with vascular endothelial cells (mediated by stem cell factor, SCF) that is critical to maintain stem cell self-renewal (Cucco *et al*., 2020; Oh *et al*., 2020). It has been postulated that this process of self-renewal enables the maintenance of a population of undifferentiated (stem) cells that enable pulp regeneration throughout the life of the dentin-pulp complex (Cucco *et al*., 2020). The multipotency of stem cells from the dental pulp has been demonstrated unequivocally by their ability to differentiate in several cell types, including odontoblasts, osteoblasts, adipocytes, neural cells, chondrocytes, and endothelial cells (Bento *et al*., 2013; Cordeiro *et al*., 2008; D’Alimonte *et al*., 2011; Gronthos *et al*., 2000; Lambrichts *et al*., 2017; Miura *et al*., 2003; Sakai *et al*., 2010; Smith *et al*., 2016; Monache *et al*., 2019; Yusof *et al*., 2018).

Vascular Endothelial Growth Factor (VEGF) is a major regulator of angiogenesis and vasculogenesis in development, maintenance of health and in disease (Apte *et al*., 2019). It has been demonstrated that VEGF induces differentiation of pulp stem cells into vascular endothelial cells via signaling through VEGF receptor 1 (VEGFR1) (Sakai *et al*., 2010; Bento *et al*., 2013; Gorin *et al*., 2016; Zhang *et al*., 2016) and induces anastomosis of these stem cell-derived blood vessels with the host vasculature via VE-Cadherin (Sasaki *et al*., 2020). Three to 5 days after induction with VEGF, dental pulp stem cells acquire VEGFR2 expression (Sasaki *et al*., 2020), which then drives vessel maturation and functional angiogenesis (Janebodin *et al*., 2013; Monache *et al*., 2019; Xu *et al*., 2019). These pulp stem cells have been considered good candidates for bone tissue engineering, as they are able to differentiate into both, vasculogenic endothelial cells and bone-forming osteoblasts when implanted in environments conducive to bone formation (D’aquino *et al*., 2007; D’aquino *et al*., 2009; Giuliani *et al*., 2013; Paino *et al*., 2017; Yusof *et al*., 2018). Interestingly, VEGF can be produced by osteoblasts in response to Bone Morphogenetic Proteins (BMP) via processes that couple angiogenesis to bone formation (Wang *et al*., 1997; Deckers *et al*., 2002).

It is well known that VEGF signals through VEGFR1 to activate MEK1/ERK signaling, inhibit STAT3 transcriptional activity, and enable endothelial differentiation of pulp stem cells (Bento *et al*., 2013). However, it is not known if all pulp stem cells express VEGFR1 and are capable of endothelial differentiation, or if only a subpopulation of pulp stem cells express VEGFR1 and therefore are “primed” for vasculogenic differentiation. Here, we unveiled a sub-population of VEGFR1-expressing pulp stem cells that is primed to undergo vasculogenic differentiation, and began to define the polylithic nature of these tissue-specific stem cells.

## Materials and Methods

### Cell culture

DPSC (Lonza, Walkersville, MD, USA) and SHED (kindly provided by Songtao Shi) were cultured in Alpha-Minimal Essential Media (Alpha-MEM; Invitrogen, Carlsbad, California) supplemented with 20% Fetal Bovine Serum (FBS; Thermo Fisher Scientific, Waltham, MA, USA), 1% Antimycotic and Antibiotic Solution (Gibco, Grand Island, NY, USA) at 37°C and 5% CO₂. Primary Human Dermal Microvascular Endothelial Cells (HDMEC; Lonza, Walkersville, MD, USA) were cultured in Endothelial Cell Growth Medium-2 (EGM2-MV; Lonza). To induce vasculogenic differentiation, SHED or DPSC were exposed to EGM2-MV supplemented with 50 ng/mL rhVEGF_165_ (R&D Systems, Minneapolis, MN, USA). In selected experiments, cells were exposed to 0 or 25 ug/mL anti-VEGF antibody, *i.e*. bevacizumab (Genentech, San Francisco, CA, USA).

### Semi-quantitative RT-PCR

Total RNA from SHED or DPSC was isolated using the Trizol (Invitrogen) method, quantified by NanoDrop (Thermo Fisher Scientific), and reverse transcribed into DNA using Superscript II Reverse Transcriptase (Invitrogen) according to the manufacturer’s instructions. The PCR primers used here were: VEGFR-1(sense 5’-actcccttgaacacgagagttc-3’, antisense 5’-gatttctcagtcgcaggtaacc-3’), VEGFR-2 (sense 5’-gctgtctcagtgacaaacccat-3’, antisense 5’-ctcccacatggattggcagagg-3’), Tie-2 (sense 5’tacacctgcctcatgctcag-3’, antisense 5’-gcagagacatccttggaagc-3’), CD31 (sense 5’-tactcagtcatggccatggt-3’, antisense 5’-ttggccttggctttcctcag-3’), VE-cadherin (sense 5’-cctggtataacctgactgtg-3’, antisense 5’-tgtgatggtgaggatgcaga-3’), and GAPDH (sense 5’-gaccccttcattgacctcaact-3’, antisense 5’-caccaccttcttgatgtcatc-3’).

### Cell sorting by flow cytometry

Cells were harvested into FACS tubes (Corning, Glendale, AZ, USA) at a density of 10^6^ cells/tube, washed with PBS and incubated with 14 μL of anti-human VEGFR1 antibody (PE-conjugated; R&D Systems) in the dark at room temperature for 35 minutes. Then, cells were washed in PBS and resuspended in Stain Buffer (BD Bioscience, San Jose, CA, EUA). Cells were sorted according to VEGFR-1 expression levels (VEGFR1^high^ or VEGFR1^low^). As negative controls, untreated cells or cells exposed to isotype-matched non-specific IgG antibody (R&D Systems). The analysis of the data was performed using the FlowJo Software (Ashland, OR, USA).

### Sulforhodamine B (SRB) assay

Sorted VEGFR1^high^ and VEGFR1^low^ cells were seeded at the density 2.5×10^3^cell/well in 96-well plates. After 24–72 hours, cells were fixed with 10% trichloroacetic acid at 4°C for 1 hour. Then, cells were washed, dried, stained with 0.4% SRB (Thermo Fisher Scientific), and incubated at room temperature for 30 minutes. To remove the unbound excess dye, cells were washed in 1% acetic acid, allowed to dry, and then the dye was solubilized with trizma-base. Plates were read in a microplate reader (GENios; Tecan, Männedorf, Switzerland) at 565 nm. Data were obtained from 8 wells per condition and time point. Here, and throughout this manuscript, experiments were performed 3 independent times to verify the reproducibility of the data.

### Immunofluorescence assay

For immunofluorescence, 5×10^4^ cells were cultured in Nunc Lab-Tek chamber slides (Millipore Sigma, Burlington, MA, USA) for 24 hours. The cells were washed and incubated overnight with rabbit anti-human VEGFR1 polyclonal antibody (Santa Cruz Biotechnology, Santa Cruz, CA, USA). Next day, excess primary antibody was washed and goat anti-rabbit antibody conjugated to Alexa Fluor 488 (Life Technologies, Eugene, OR, USA) was used to visualize VEGFR1. Nuclei were stained with Vectashield Mounting Medium containing DAPI (Vector Laboratories, Burlingame, CA, USA).

### Western blot

DPSC or SHED cells were lysed in NP-40 and protein concentration was quantified at 595 nm (GENios, Tecan). Protein lysates underwent electrophoresis and transferred to nitrocellulose membranes that were blocked in 5% milk for 30 minutes. Membranes were exposed overnight at 4°C to the following primary antibodies: anti-human VEGFR-1, VEGFR-2, Tie-2, CD31, VE-Cadherin (Santa Cruz Biotechnology, Santa Cruz, CA, USA) or GAPDH (MiliporeSigma, Burlington, MA, USA) in the following dilutions 1:1000, 1:1000, 1:1000, 1:3000, 1:3000, 1:4×10^7^, respectively. Next day, membranes were washed 2X in TBST, incubated with appropriate secondary antibodies for 2 hours, washed again, and exposed to SuperSignal West Pico chemiluminescent substrate (Thermo Fisher Scientific).

### *In Vitro* vasculogenic differentiation assay

2×10^5^ cells flow sorted as VEGFR1^high^ or VEGFR1^low^ were plated in standard tissue culture plates and cultured in alpha-MEM supplemented with 20% FBS. Next day, vasculogenic differentiation medium (EGM2-MV supplemented with 50 ng/mL rhVEGF_165_) was added for up to 9 days. To confirm the endothelial differentiation of the dental pulp stem cells, western blots were performed for VEGFR2, CD-31, VE-cadherin, and Tie-2, as we showed (Bento *et al*., 2013; Sasaki *et al*., 2020).

### *In Vitro* capillary sprouting assay

After sorting for VEGFR1, 10⁴ cells/well were seeded in 12-well plates pre-coated with Growth Factor Reduced Matrigel (BD Bioscience, Bedford, MA, USA) and cultured for up to 10 days in vasculogenic differentiation medium, i.e. (EGM2-MV supplemented with 50 ng/mL rhVEGF_165_) in presence of 0 or 25 *μ*g/mL anti-human VEGF antibody (bevacizumab; Genentech, CA, USA). Capillary sprouts were counted under a light microscope at 100x magnification in 12 random fields per well in 3 independent wells per experimental condition.

### *In vivo* vasculogenic differentiation assay

Biodegradable, highly porous, poly-L-lactic acid (PLLA) scaffolds were prepared and were cut in 6 mm × 6 mm × 1 mm, as described (Nör *et al*., 2001). SHED cells (6×10^5^ cells/scaffold) flow sorted for VEGFR1 were mixed with Matrigel, seeded in the scaffolds (n=6), and transplanted into the subcutaneous space of the dorsum of severe combined immunodeficient mice (CB-17 SCID; Jackson Laboratory, Bar Harbor, ME, USA). After 28 days, scaffolds were retrieved and fixed in 10% formaldehyde for 24 hours at 4°C. Histologic sections (5-μm-thick) were stained with hematoxylin-eosin or kept unstained for immunohistochemistry assay. Tissue sections were deparaffinized, antigen retrieval was performed with 1mg/ml Trypsin (Merck, Darmstadt, Germany) for 1 hour at 37° C. After incubation in 0.1% Triton-X-100 and 3% H_2_O_2_, and background Sniper (Biocare Medical, Pacheco, CA, USA) for 20 minutes at room temperature, tissue sections were incubated in 1:100 rabbit anti-human CD31 (Bethyl Laboratories, Montgomery, TX, USA) or rabbit anti-Factor VIII (Ab-1; Thermo Fisher Scientific). Next day, unbound primary antibodies were washed with Wash Buffer (Dako, Carpinteria, CA, USA) and MACH3 Rabbit/Mouse Probe (Biocare Medical), MACH 3 Rabbit/Mouse HRP-Polymer (Biocare Medical), Betazoid DAB Chromogen Kit (Biocare Medical) were added to the tissue sections for 20 minutes each, except for the DAB incubation that was performed 1–2 minutes. After the final wash, VectaMount (Vector Laboratories, Burlingame, CA, USA) was added for cover slip placement. Human microvessels (CD31-positive) were counted in 8 random fields (200x) by a calibrated researcher blinded for experimental conditions using the Image J software (NIH). The animal work included here was done under a protocol (PRO00009087) approved by the University of Michigan Animal Ethics Committee.

### Statistical analysis

The statistical analyses were performed using the GraphPad Prism software (GraphPad, San Diego, CA, USA). The Shapiro-Wilk normality test was applied in the quantitative measurements. Data were analyzed by t-tests or one-way ANOVA followed by appropriate post-hoc tests. The significance was set at p<0.05.

## Results

### Baseline expression of VEGFR1 and VEGFR2 in dental pulp stem cells

**Fig. 1.**
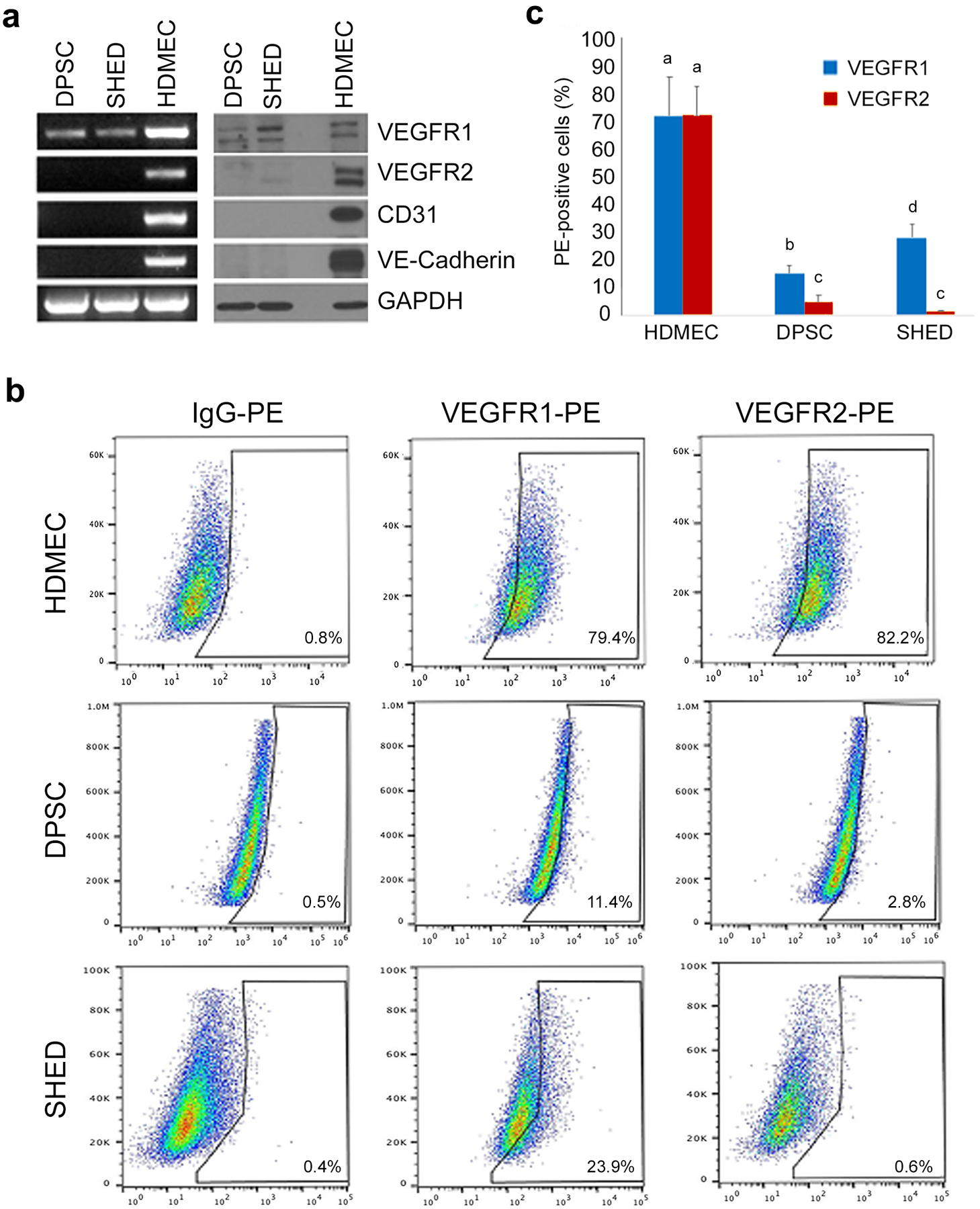
Baseline expression of VEGFR1 and VEGFR2 in dental pulp stem cells. **(a)** RT-PCR and Western Blot analyses of VEGFR1, VEGFR2, CD-31, and VE-cadherin expression in DPSC and SHED cultured in alpha-MEM + 20% FBS. **(b)** Flow cytometric analyses of VEGFR1 and VEGFR2 expression in SHED, DPSC and human dermal microvascular endothelial cells (HDMEC). Cells are presented in a dot plot of Side Scatter Area (SSC-A) gating against PE fluorescence. Cells were analyzed using anti-VEGFR1 and anti-VEGFR2 PE-conjugated antibodies, and an isotype-matched IgG as control to set the gating. **(c)** Graph depicting the percentage of VEGFR1 and VEGFR2-positive cells in SHED, DPSC and HDMEC. Different low case letters indicate statistical significance at p<0.05.

To evaluate the baseline level of expression of key mediators of vasculogenesis in pulp stem cells, RT-PCR (mRNA) and Western blots (protein) of untreated DPSC and SHED cells were performed, and primary human endothelial cells (HDMEC) were used as controls. While endothelial cells expressed all markers evaluated here (i.e. VEGFR1, VEGFR2, CD31 and VE-Cadherin), SHED and DPSC cells only expressed VEGFR1 at baseline (Figure 1a). An intrinsic limitation of both RT-PCR and Western blots is the fact that cells are pooled together for these assays, which does not allow for the understanding of expression levels of these markers in individual cells. To overcome this limitation, these cells were analyzed by flow cytometry for VEGFR1 or VEGFR2 (Figure 1b,c). We observed that approximately 10% and 20% of DPSCs and SHED cells express VEGFR1, respectively. In contrast, only a negligeable percentage of DPSC and SHED express VEGFR2. These data are consistent with the results obtained in the RT-PCR and Western blots, and suggest that VEGFR1 (not VEGFR2) is the receptor engaged by VEGF to induce the vasculogenic differentiation of pulp stem cells.

### VEGFR1 does not regulate proliferation of pulp stem cells

**Fig. 2.**
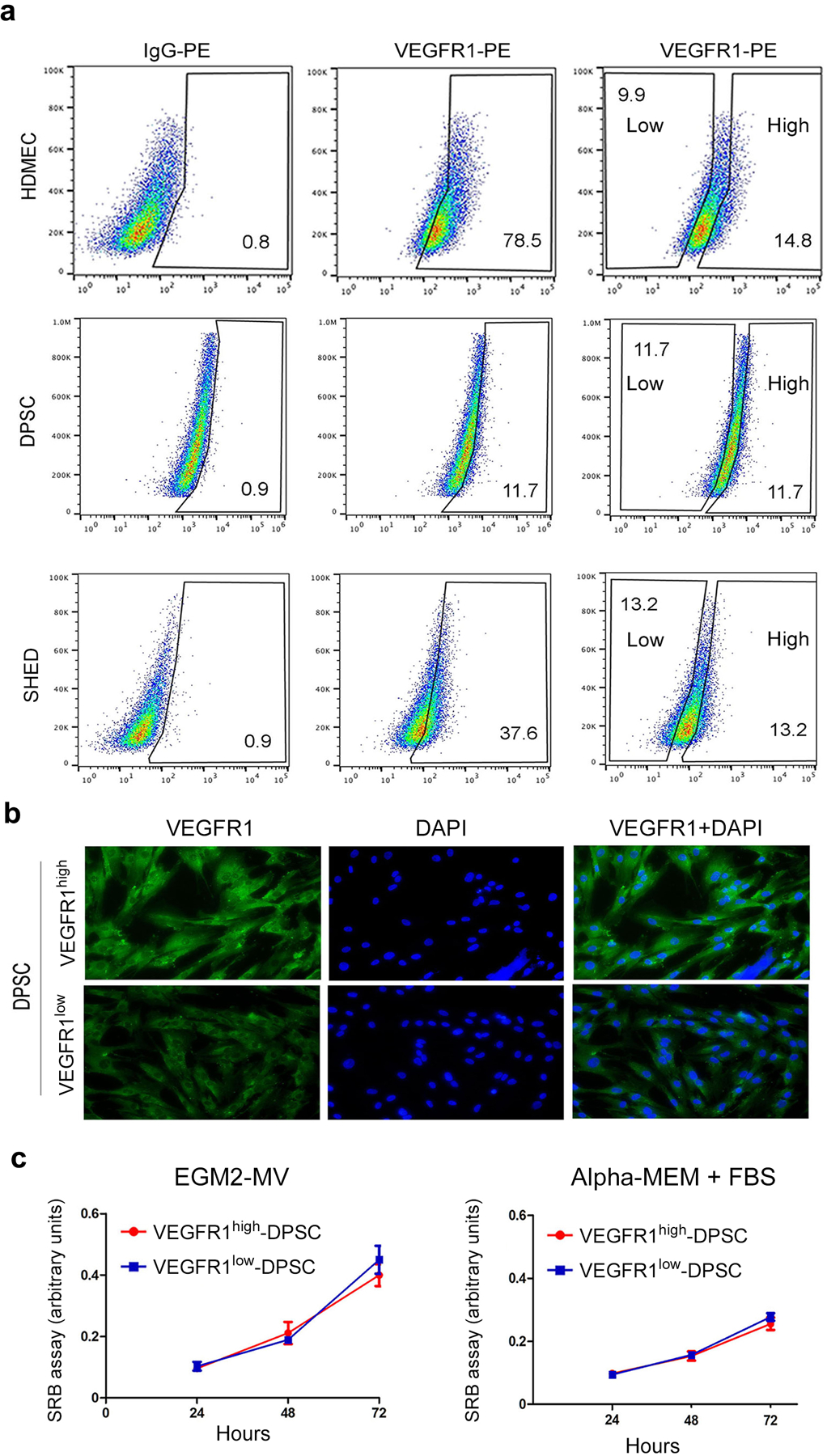
VEGFR1 does not regulate proliferation of pulp stem cells. **(a)** Flow sorting of HDMEC, SHED, and DPSC according to VEGFR1 expression levels (*i.e*. high and low), using isotype-matched IgG to set the gates. For DPSC and SHED, we sorted out equivalent percentages of VEGFR1^high^ and VEGFR1^low^ cells. **(b)** Fluorescence microscopy images of VEGFR1^high^ and VEGFR1^low^ cells. Green depicts VEGFR1 expression while blue depicts DAPI nuclear staining. bar=20 μm. **(c)** Line graph depicting cell proliferation over time when VEGFR1^high^ and VEGFR1^low^ cells, as determined by the SRB assay. Cells were cultured in vasculogenic differentiation medium (EGM2-MV + 50 ng/ml rhVEGF_165_) or alpha-MEM + 20% FBS for 24 to 72 hours. Data represents average +/− s.d. in 8 wells per condition.

To examine the impact of VEGFR1 expression levels on the proliferation rate, flow sorting was used to generate a subpopulation of VEGFR1^high^ pulp stem cells and a subpopulation of VEGFR1^low^ pulp stem cells (Figure 2a). Immunofluorescence analysis showed that both populations, VEGFR1^high^ and VEGFR1^low^ cells, exhibited a homogeneous distribution of VEGFR1 expression (Figure 2b). Interestingly, the level of VEGFR1 expression (i.e. high or low) had no impact in cell density (surrogate for net effect of treatment on cell proliferation and cell survival), when DPSC cells were cultured in either basal culture medium (*i.e*. AlphaMEM + FBS) or in vasculogenic differentiation medium (i.e. EGM2-MV + 50 ng/ml rhVEGF_165_) (Figure 2c).

### VEGFR1^high^ pulp stem cells are more vasculogenic than VEGFR1^low^ cells *in vitro*

**Fig. 3.**
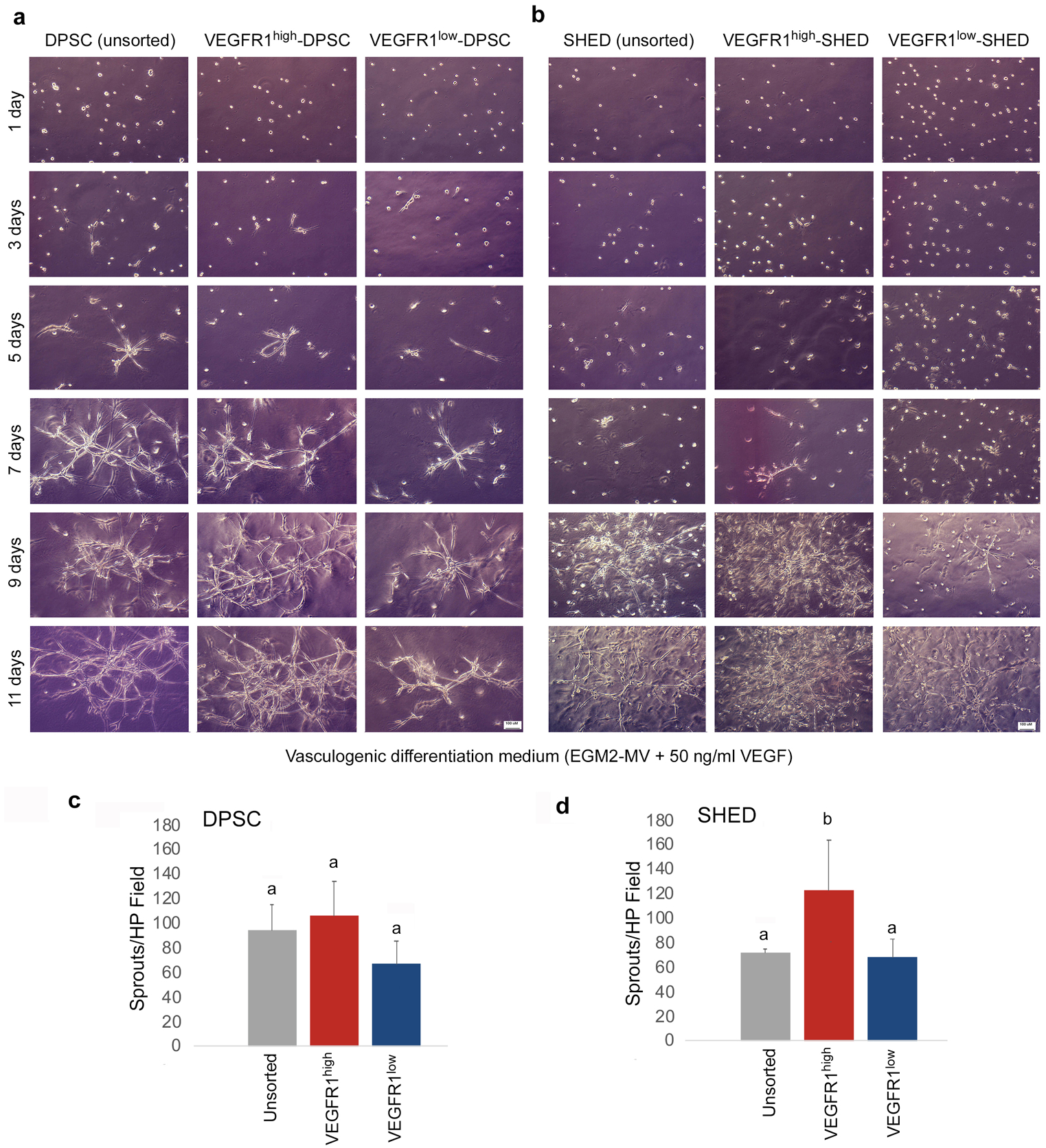
VEGFR1^high^ pulp stem cells generate more capillary sprouts than VEGFR1^low^ cells *in vitro*. Cells were sorted for VEGFR1 expression levels and then plated in plates coated with growth factor reduced Matrigel. (**a,b**) Representative photomicrographs (bar: 100 μm) of capillary sprouts generated by VEGFR1^high^, VEGFR1^low^, or unsorted DPSC and SHED cells cultured in vasculogenic differentiation medium for up to 11 days. (**c,d**) Bar graphs showing the number of capillary-like sprouts at the end of the experimental period (*i.e*. 11 days). Different low case letters indicate statistical significance at p<0.05. Number of capillary sprouts (average +/− s.d.) is representative of 12 random microscopic fields from triplicate wells per condition.

**Fig. 4.**
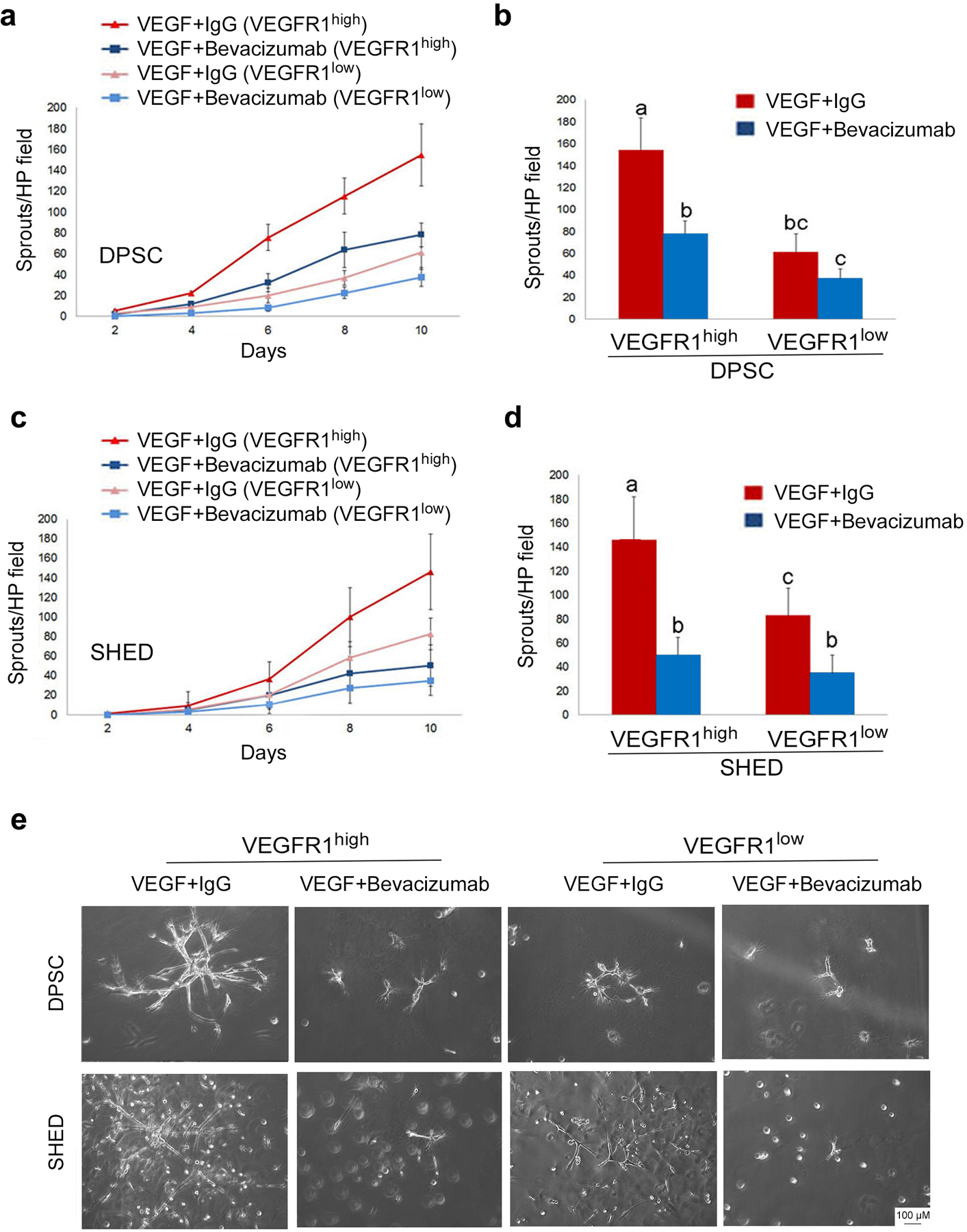
VEGF blockade inhibits the vasculogenic potential of VEGFR1^high^ cells *in vitro*. **(a,c)** Line graphs depicting the number of sprouts per high power field generated by DPSC or SHED. **(b,d)** Bar graphs showing the number of capillary-like sprouts at the end of the experimental period (i.e. 10 days). VEGFR1^high^ and VEGFR1^low^ DPSC or SHED cells were cultured in wells pre-coated with growth factor reduced Matrigel and stimulated with vasculogenic differentiation medium in presence of 0 or 25 μg/ml bevacizumab (anti-VEGF antibody). Different low case letters indicate statistical significance at p<0.05. Number of capillary sprouts (average +/− s.d.) is representative of 12 random microscopic fields from triplicate wells per condition. **(e)** Representative photomicrographs of the capillary sprouts observed after 10 days under the experimental conditions described above (bar: 100 μm).

To begin to evaluate the impact of VEGFR1 expression on the vasculogenic potential of dental pulp stem cells, DPSC and SHED were sorted for VEGFR1 levels, plated the cells in Matrigel-coated wells, and exposed them to vasculogenic differentiation medium for 11 days. The images depicted here (representative of 3 independent experiments) showed that VEGFR1^high^ SHED cells are more vasculogenic than VEGFR1^low^ SHED (Figure 3). A similar trend was observed when DPSC cells were analyzed under similar experimental conditions (Figure 3). To verify the specificity of these results, an independent set of studies in which sorted SHED and DPSC were exposed to vasculogenic differentiation medium in presence (or not) of an anti-VEGF antibody (bevacizumab) was performed (Walker *et al*., 2012). These experiments demonstrated that VEGFR1^high^ DPSC cells generate more capillary sprouts than VEGFR1^low^ DPSC cells (Figure 4a,b). The same trends were observed with VEGFR1^high^ SHED cells versus VEGFR1^low^ SHED cells (Figure 4c,d). Notably, blockade of VEGF with bevacizumab decreased the number of capillary sprouts generated by DPSC and SHED (Figure 4a–e), demonstrating that the responses observed here were dependent upon active VEGF signaling.

### VEGFR1^high^ pulp stem cells are more vasculogenic than VEGFR1^low^ cells *in vivo*

**Fig. 5.**
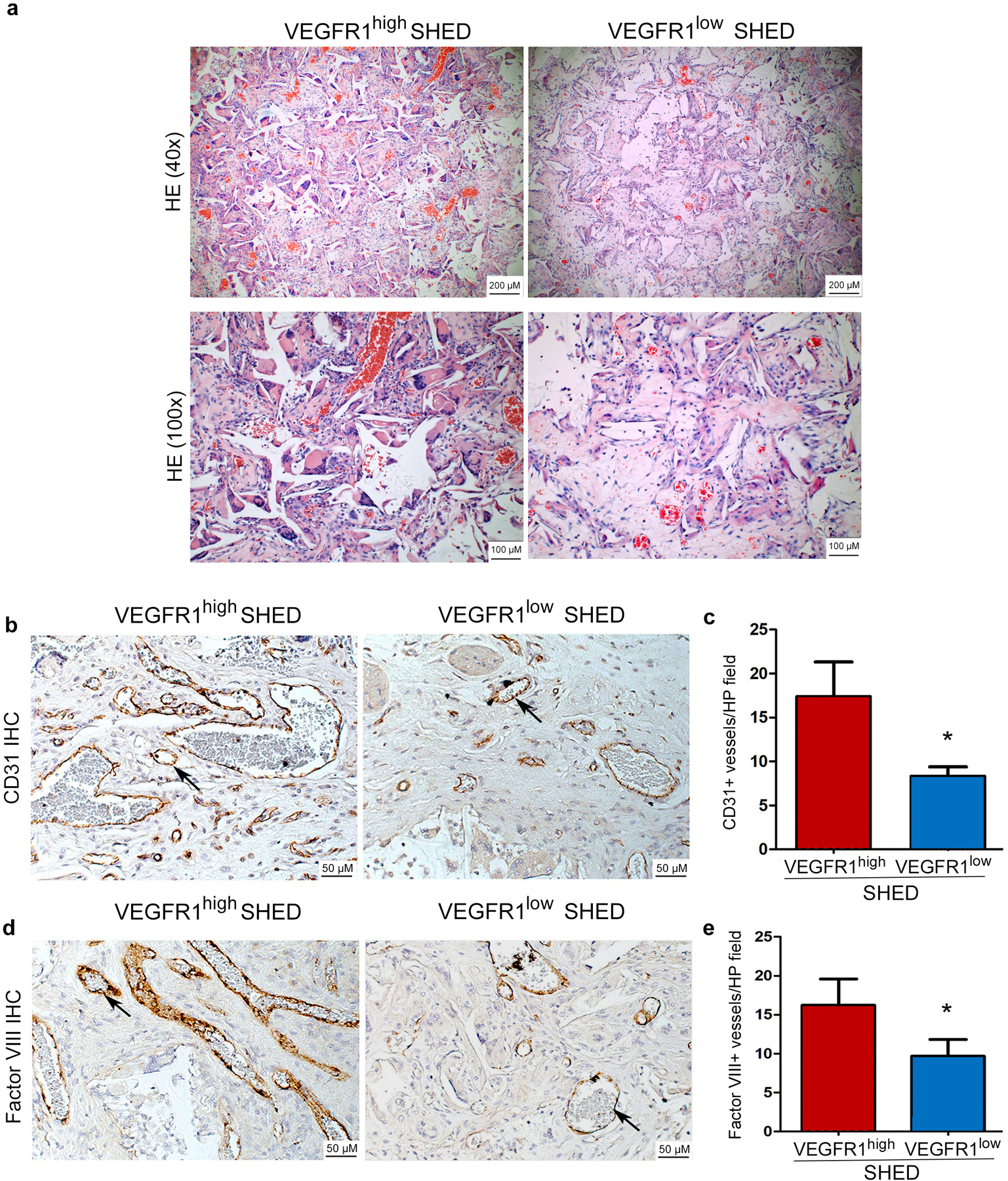
VEGFR1^high^ SHED cells are more vasculogenic than VEGFR1^low^ SHED *in vivo*. **(a,c)** Human VEGFR1^high^ and VEGFR1^low^ SHED were seeded in biodegradable scaffolds (n=6 per experimental condition) and transplanted into the subcutaneous space of immunodeficient mice. Four weeks after transplantation, the scaffolds were retrieved, fixed, and paraffin embedded. (a) representative images of sections stained with Hematoxylin and eosin at low and high magnification (bar: 100 μm/ 200 μm) and (**b,d**) Immunohistochemistry with anti-human CD-31 or anti-Factor VIII antibody to identify blood vessels (brown color). Representative vessels are highlighted with black arrows (bar: 50 μm). **(c,e)** Graphs depicting the number of CD31-positive or Factor VIII-positive blood vessels inside the scaffolds. Data represent analysis of 8 randomly selected microscopic fields from each scaffold (n=6) at 200x.

Considering that SHED and DPSC presented similar results in the *in vitro* studies performed here (cell proliferation, capillary-like sprouting and response to therapeutic blockade of VEGF signaling with bevacizumab), a decision was made to focus on the use of SHED as model pulp stem cells for the *in vivo* studies. To understand the impact of VEGFR1 expression on the vasculogenic potential of dental pulp stem cells, SHED cells were sorted for VEGFR1, seeded in biodegradable scaffolds, and transplanted into SCID mice, as shown (Bento *et al*., 2013; Nör *et al*., 2001; Sasaki *et al*., 2020). Similar to *in vitro* experiments, VEGFR1^high^ SHED are more vasculogenic than VEGFR1^low^ cells in 8 randomly selected high-power fields per scaffold (n=6) (Figure 5). Using the anti-human CD31 antibody, which is specific to human endothelial cells (Nör *et al*., 2001), we observed that scaffolds seeded with VEGFR1^high^ SHED contained approximately twice as many blood vessels as scaffolds seeded with VEGFR1^low^ SHED cells (Figure 5a,b). Notably, immunohistochemistry with anti-Factor VIII antibody confirmed the results obtained with anti-CD31 (Figure 5c,d), despite the fact that the anti-Factor VIII antibody used here crossreact with both human and mouse endothelial cells. These findings confirm our previous reports that transplantation of human endothelial cells or human dental pulp stem cells result in the engineering of human blood vessels in murine hosts (Bento *et al*., 2013; Nör *et al*., 2001; Sakai *et al*., 2010; Sasaki *et al*., 2020).

## Discussion

Dental pulps stem cells are rather unique stem cells developmentally derived from the neural crest (REF). The 2 major hallmarks of physiological stemness (multipotency and self-renewal) have been extensively characterized in these pulp stem cells (Cucco *et al*., 2020; Gronthos *et al*., 2000; Lambrichts *et al*., 2017; Miura *et al*., 2003; Oh *et al*., 2020; Sakai *et al*., 2010). While it is well-known that dental pulp stem cells can differentiate into multiple cell types, it is unclear whether every single stem cell is multipotent or if dental pulp stem cells are a heterogeneous cell type containing smaller sub-groups of cells that are “primed” to undergo diverse differentiation pathways. Here, we began to explore this question by hypothesizing that stem cells of dental pulp origin contain a subgroup of cells that are primed to undergo a vasculogenic differentiation pathway.

VEGFR1 and VEGFR2 are constitutively expressed in endothelial cells and function as the primary regulators of VEGF signaling in blood vessels (Karaman *et al*., 2018; Trapiella-Alfonso *et al*., 2018). While VEGFR1 signaling is required for the survival of vascular endothelial cells, VEGFR2 regulates blood vessel sprouting and neovascularization (Zhang *et al*., 2010). Interestingly, the receptor that fine tune angiogenesis and vascular remodeling is VEGFR2, but VEGF binds to VEGFR1 (soluble or membrane bound) with more affinity than to VEGFR2. In this way, the number of VEGF molecules available to bind to VEGFR2 is modulated and the angiogenic process is regulated (Balsera *et al*., 2017; Millauer *et al*., 1993; Trapiella-Alfonso *et al*., 2018). We observed here that dental pulp stem cells express VEGFR1 constitutively, but not VEGFR2. However, VEGFR2 expression can be induced upon exposure of dental pulp stem cells to vasculogenic differentiation medium containing VEGF_165_ (Bento *et al*., 2013; Sasaki *et al*., 2020). We also observed that expression of CD31 and VE-Cadherin follow upregulation of VEGFR2 expression in dental pulp stem cells (Sasaki *et al*., 2020). Indeed, VEGF induces activation of MEK/ERK signaling and induction of ERG transcriptional activity that results in expression of VE-Cadherin (Sasaki *et al*., 2020). Collectively, these data suggest that VEGF binding to VEGFR1 initiates the vasculogenic differentiation of dental pulp stem cells. Once these cells begin to express VEGFR2, then they acquire the capacity of becoming a differentiated vascular endothelial cell expressing CD31 that is able of forming functional vascular networks that anastomize with existing vessels through the function of VE-Cadherin (Sasaki *et al*., 2020).

An important issue to consider here is the scope of the impact of VEGF signaling through VEGFR1 on vasculogenic responses mediated by dental pulp stem cells. It is known that VEGF induces proliferation, migration and survivals of endothelial cells, but these cells express both VEGFR1 and VEGFR2 (Apte *et al*., 2019; Karaman *et al*., 2018). However, the full impact of VEGF on dental pulp stem cells (expressing only VEGFR1 at baseline) was unclear. The results presented here demonstrated that VEGFR1 levels (*i.e*. high or low) had no impact on DPSC proliferation when cells were exposed to vasculogenic medium (containing 50 ng/ml VEGF_165_) or regular medium (containing trace levels of VEGF present in bovine serum). As such, the increased number of capillary sprouts observed here with high VEGFR1 cells is not simply a consequence of increased number of cells. Our *in vitro* data also suggest that in unsorted conditions the VEGFR1^high^ population “takes over” and exhibit a predominant effect on overall capillary sprouting, as the number of sprouts generated by unsorted cells is lower than that of sorted VEGFR1^high^ cells (particularly with SHED cells).

Western blots and flow cytometric analyses demonstrated that a higher percentage of SHED cells exhibit high levels of VEGFR1, when compared to DPSC cells. This is consistent with the observation that SHED cells are more angiogenic than DPSC in response to VEGF (Xu *et al*., 2018), and with the results of the capillary sprout assays performed here with unsorted cells. However, once we sorted out the VEGFR1^high^ cells from both DPSC and SHED, the sorted cells from both cell types generated similar numbers of capillary sprouts *in vitro*. As such, one concludes that the vasculogenic potential of each individual VEGFR1^high^ SHED is similar to the vasculogenic potential of each individual VEGFR1^high^ DPSC. But, in aggregate SHED are more vasculogenic because they contain about twice as many VEGFR1^high^ cells as DPSC from permanent teeth.

For many years, our laboratory worked under the assumption that dental pulp stem cells were monolithic, *i.e*. they consisted of a homogeneous cell population in which multipotency was a consequence of the possibility of each stem cell to differentiate into several different cell types (Graphical Abstract). However, a series of observations contradict this hypothesis, at least in regards to vasculogenic differentiation, as follows. Studies performed several years ago demonstrated that global (shRNA-mediated) silencing of VEGFR1 expression inhibits vasculogenic differentiation of dental pulp stem cells (Bento *et al*., 2013; Sakai *et al*., 2010). However, at that time we did not know if every single dental pulp stem cell expressed VEGFR1, or if only a sub-population of these cells expressed VEGFR1 (and was capable of responding to VEGF stimulation). Here, we observed that only 10–15% DPSC (permanent teeth) and 20–25% SHED (primary teeth) express constitutive VEGFR1, while the remaining cells (*i.e*. the majority of these cells) do not express this receptor. This finding gave rise to the hypothesis that dental pulp stem cells constitute of polylithic (*i.e*. heterogeneous) cells containing one small sub-population of cells that are primed to respond to VEGF stimulation and undergo vasculogenic differentiation (as they express VEGFR1), while the remaining cells cannot respond to VEGF (as they do not express VEGFR1). This raises the intriguing possibility that other sub-populations of dental pulp stem cells are primed to undergo alternative differentiation pathways, such as odontoblastic or neurogenic fates (Graphical Abstract). We are currently pursuing studies that aim at expanding the understanding of the polylithic hypothesis, through identification of signaling events and characterization of dental pulp stem cells that undergo non-vasculogenic differentiation pathways.

A limitation inherent to our study design is that we do not know the stability of VEGFR1 expression levels after transplantation of the cells into murine hosts. It is possible that cells that were initially sorted as VEGFR1^high^ cells do not maintain a high VEGFR1 expression level after several weeks in the mouse, and conversely if the VEGFR1^low^ cells remain exhibiting low expression levels of this receptor. These expression levels cannot be accurately quantified in the SHED-derived blood vessels *in vivo*. Notably, this perceived limitation may explain the observation that SHED-derived blood vessels were also found in scaffolds seeded with VEGFR1^low^ cells, albeit in significantly lower numbers.

In conclusion, this work demonstrates the critical role of VEGF signaling through VEGFR1 for the vasculogenic differentiation of dental pulp stem cells. Perhaps more importantly, it demonstrates that dental pulp stem cells are polylithic and contain at least one unique subset of stem cells characterized by high VEGR1 expression that are primed for vasculogenic differentiation. These results suggest the possibility of purifying specific subpopulations of pulp stem cells according to specific needs. This discovery raises the possibility of sorting for, or specifically engaging, VEGFR1^high^ dental pulp stem cells for vascular engineering and treatment of ischemic conditions.
